# Member species of the *Anopheles gambiae* complex can be misidentified as *Anopheles leesoni*

**DOI:** 10.1186/s12936-020-03168-x

**Published:** 2020-02-24

**Authors:** Yael Dahan-Moss, Allison Hendershot, Minishca Dhoogra, Henry Julius, Jacek Zawada, Maria Kaiser, Neil F. Lobo, Basil D. Brooke, Lizette L. Koekemoer

**Affiliations:** 1 Centre for Emerging Zoonotic and Parasitic Diseases, National Institute for Communicable Diseases of the National Health Laboratory Service, Johannesburg, South Africa; 2 Wits Research Institute for Malaria, School of Pathology, Faculty of Health Sciences, University of the Witwatersrand, Johannesburg, South Africa; 3 Eck Institute for Global Health, University of Notre Dame, Notre Dame, IN USA

**Keywords:** Species identification, *Anopheles leesoni*, *Anopheles gambiae* multiplex PCR, *An. funestus* multiplex PCR, Morphology, Dichotomous keys

## Abstract

**Background:**

Accurate *Anopheles* species identification is key for effective malaria vector control. Identification primarily depends on morphological analysis of field samples as well as molecular species-specific identifications. During an intra-laboratory assessment (proficiency testing) of the *Anopheles funestus* group multiplex PCR assay, it was noted that *Anopheles arabiensis* can be misidentified as *Anopheles leesoni*, a zoophilic member of the *An. funestus* group. The aim of this project was, therefore, to ascertain whether other members of the *Anopheles gambiae* complex can also be misidentified as *An. leesoni* when using the standard *An. funestus* multiplex PCR.

**Methods:**

The *An. funestus* multiplex PCR was used to amplify DNA from *An. gambiae* complex specimens. These included specimens from the laboratory colonies and field samples from the Democratic Republic of Congo. Amplified DNA from these specimens, using the universal (UV) and *An. leesoni* species-specific primers (LEES), were sequence analysed. Additionally, *An. leesoni* DNA was processed through the diagnostic *An. gambiae* multiplex PCR to determine if this species can be misidentified as a member of the *An. gambiae* complex.

**Results:**

Laboratory-colonized as well as field-collected samples of *An. arabiensis*, *An. gambiae*, *Anopheles merus*, *Anopheles quadriannulatus, Anopheles coluzzii* as well as *Anopheles moucheti* produced an amplicon of similar size to that of *An. leesoni* when using an *An. funestus* multiplex PCR. Sequence analysis confirmed that the UV and LEES primers amplify a segment of the ITS2 region of members of the *An. gambiae* complex and *An. moucheti*. The reverse was not true, i.e. the *An. gambiae* multiplex PCR does not amplify DNA from *An. leesoni*.

**Conclusion:**

This investigation shows that *An. arabiensis*, *An. gambiae*, *An. merus*, *An. quadriannulatus, An. coluzzii* and *An. moucheti* can be misidentified as *An. leesoni* when using *An. funestus* multiplex PCR. This shows the importance of identifying specimens using standard morphological dichotomous keys as far as possible prior to the use of appropriate PCR-based identification methods. Should there be doubt concerning field-collected specimens molecularly identified as *An. leesoni*, the *An. gambiae* multiplex PCR and sequencing of the internal transcribed spacer 2 (ITS2) can be used to eliminate false identifications.

## Background

Malaria is a major vector borne disease that is most prevalent in sub-Saharan Africa. There were approximately 213 million cases and 380,000 malaria-related deaths in this region in 2018, accounting for 93% of cases and 94% of deaths from malaria reported globally [1].

A key component of malaria control is suppression of *Anopheles* mosquito vectors.

The primary methods used for malaria vector control are indoor residual spraying (IRS) of formulated insecticides, insecticide-treated nets (ITN) and larval source management (LSM) [2]. These can be incorporated into broader, tailored strategies within an integrated vector management (IVM) framework [3]. Other initiatives under development include attractive toxic sugar baits (ATSB), spatial repellents, housing improvements, endectocide use and genetic approaches [4–8].

The major malaria vector mosquito species in Africa are *Anopheles gambiae*, *Anopheles arabiensis* and *Anopheles coluzzii* of the *An. gambiae* species complex, and *Anopheles funestus* of the *An. funestus* species group [9–12]. In addition to these, other species within these taxa—including *Anopheles merus* of the *An. gambiae* complex, and *Anopheles rivulorum*, *Anopheles parensis*, *Anopheles vaneedeni* and *Anopheles leesoni* of the *An. funestus* group—have been implicated as secondary malaria vectors at various African localities [11, 13–23] to mention but a few. Importantly, primary and secondary vector species often occur in sympatry in varying combinations depending on locality [11], different species may display different behaviours, such as indoor or outdoor feeding and resting [21, 24], and may vary in their susceptibilities to insecticide [19, 25–27]. It is, therefore, necessary to identify the entomological drivers of localized malaria transmission by using tailored vector surveillance strategies. These include judicious use of sampling techniques followed by species identifications, vector incrimination (sporozoite detection) and insecticide susceptibility assessments of these populations. The information generated in this way provides the necessary baseline data needed to guide control interventions that target incriminated vector populations based on their specific traits, such as their resting and feeding preferences (indoor vs. outdoor), their preferred breeding sites (perennial vs. temporary) and their insecticide susceptibilities. The same surveillance techniques can also be used to assess the effectiveness of interventions post implementation.

The accurate identification of malaria vector species is, therefore, central to the application of successful vector control interventions, primarily by ensuring the efficient and effective use of limited resources available to vector control programmes. Misidentification of *Anopheles* species can lead to misapplication of vector control interventions [28–30]. An example comes from Zimbabwe in the early 1970s, when *An. quadriannulatus*, a non-vector member of the *An. gambiae* complex, could not easily be distinguished from the vector *An. arabiensis*. Insecticide susceptibility tests on mixed samples of *An. quadriannulatus* and *An. arabiensis* suggested susceptibility to the insecticide dieldrin [28, 29]. What was not however evident at the time was that the samples that succumbed to dieldrin exposure were *An. quadriannulatus*, while the few survivors were *An. arabiensis*, implying resistance in the vector population. The use of dieldrin for indoor residual spraying did not therefore achieve the desired effect on malaria transmission, and the insecticide regimen was subsequently changed once accurate species identifications were used to differentiate between resistance in the *An. arabiensis* vector population and susceptibility in the *An. quadriannulatus* non-vector population [28, 29].

Identification to species of field-collected mosquito specimens depends on the use of external morphological characters followed by molecular methods where indicated [9, 10, 31]. This is especially pertinent for members of the *An. gambiae* complex and *An. funestus* group whose member species vary significantly in their behavioural traits and vector competencies. The subsequent use of diagnostic molecular procedures to identify specimens to species is required because of morphological similarities between members within each taxon [32, 33].

Morphological identification of mosquitoes can be done at district level and is not reliant on expensive molecular equipment. Subsequent molecular analysis to identify indicated specimens to species (using multiplex PCR assays) is generally conducted at established laboratories at the national level or within research institutes with sufficient capacity [34–36]. These species-specific assays are an important diagnostic tool and are regularly used in laboratories for research and routine vector surveillance [34–36]. Molecular sequencing of target genes has been used for *Anopheles* species identifications [21, 37–41]. Laboratory infrastructure and cost, however, preclude this method from being routinely used in support of vector surveillance.

Regardless of the method used for molecular species identification, quality assurance (QA) of the data produced is critical. This is because the pertinence and relevance of all follow-on associative analyses (vector incrimination/sporozoite detection, insecticide susceptibility assessments, associated behaviours) is dependent on accurate species identification. An essential requirement of QA is regular proficiency testing of laboratory staff to monitor their competency in the application of diagnostic assays [42, 43]. A recent proficiency assessment exercise conducted at the Vector Control Reference Laboratory of the National Institute for Communicable Diseases (NICD) in Johannesburg was based on an intra-laboratory comparison using the *An. funestus* multiplex PCR method [35, 36]. Unexpectedly, *An. arabiensis*, which was used as a blind negative control, produced an amplicon of similar size to that of *An. leesoni* when using the *An. funestus* PCR.

It has recently been established that specimens not of the *An. gambiae* complex or *An. funestus* group can be misidentified as members of either of these taxa by using the corresponding multiplex PCR assays in the absence or misidentification of a priori morphological identification [33]. Morphological identification on field samples can be problematic if samples are damaged due to mosquito handling (collection method, preservation processing) or due to age of the mosquito samples. Based on these data, the aim of this study was to ascertain whether *An. gambiae* complex specimens can easily be misidentified as *An. leesoni* when using the *An. funestus* multiplex PCR.

## Methods

### In silico sequence analysis of *Anopheles funestus* multiplex PCR primers and *Anopheles gambiae* complex species internal transcribed spacer 2 (ITS2) region

The sequences of primers used in the *An. funestus* multiplex PCR [35, 36] were compared with ITS2 sequences from the *An. gambiae* complex species to identify sequence similarities. Nucleotide Basic Local Alignment Search Tool (BLAST) (https://blast.ncbi.nlm.nih.gov/Blast.cgi) and Emboss Needle pairwise sequence alignment tool (https://www.ebi.ac.uk/Tools/psa/emboss_needle/nucleotide.html) were used.

### Laboratory-reared *Anopheles gambiae* complex species samples

Specimens of *An. funestus*, *An. arabiensis*, *An. gambiae*, *An. merus* and *An. quadriannulatus* (FUMOZ, KGB, COGS, MAFUS and SANGWE colonies respectively) housed in the Botha De Meillon insectary at the National Institute for Communicable Diseases in Johannesburg were used. The *An. leesoni* positive control was obtained from a field sample from Limpopo Province, South Africa, in December 2016. This sample was verified as *An. leesoni* by morphological and PCR species identification as well as ITS2 sequence analysis.

### PCR

*DNA extraction:* DNA was extracted from the *An. funestus*, *An. leesoni*, *An. arabiensis*, *An. gambiae*, *An. merus* and *An. quadriannulatus* specimens using prepGEM Insect DNA extraction kit (ZyGEM, PIN0020).

*Anopheles funestus* multiplex PCR: Each PCR reaction contained extracted DNA from *An. funestus* and *An. leesoni* positive controls; a “no DNA template” negative control (PCR master mix without DNA template); “extraction kit” negative controls (PCR master mix with extraction mix performed without mosquito sample), and extracted DNA from *An. arabiensis*, *An. gambiae*, *An. merus* and *An. quadriannulatus* specimens.

Several variations of the *An. funestus* multiplex PCR were performed during this investigation: (1) Standard *An. funestus* multiplex PCR with the annealing temperature set at 45 °C as per the protocol by Koekemoer et al. [35] and Cohuet et al. [36] or with the exception of the annealing temperature set at 50 °C; (2) Standard *An. funestus* multiplex PCR with the exception of the LEES primer being omitted from the PCR reaction, and with the annealing temperature set at 45 °C or 50 °C; (3). Standard *An. funestus* multiplex PCR with the exception of the PCR reaction only including the UV and LEES primers, and with the annealing temperature set at 45 °C or 50 °C. The different variations of the *An. funestus* multiplex PCR were used to test whether a non-specific PCR amplicon is produced while using the DNA of *An. gambiae* complex specimens in the PCR. Subsequently, the *An. funestus* multiplex PCRs with or without only the LEES reverse primer were used to establish whether this primer is responsible for amplification of DNA from *An. gambiae* complex specimens in the PCR. Different annealing temperatures were used in the PCRs to determine whether the annealing temperature reduces non-specific amplification of DNA from the *An. gambiae* complex when performing an *An. funestus* multiplex PCR.

*Anopheles gambiae* multiplex PCR: PCR was performed according to the protocol by Scott et al. [34]. The PCR reaction contained extracted DNA from *An. arabiensis*, *An. gambiae*, *An. merus* and *An. quadriannulatus* positive controls; a “no DNA template” negative control (PCR master mix without DNA template); “extraction kit” negative controls (PCR master mix with extraction mix performed without mosquito sample) and extracted DNA from an *An. leesoni* positive control.

The PCR products from the *An. funestus* and *An. gambiae* amplifications were electrophoresed on a 2.5% agarose gel and viewed with a ChemiDoc XRS + Imaging system (Biorad).

### Sequencing analysis

The *An. leesoni* sized amplicons produced by the UV and LEES primers were purified and sequenced through Macrogen (http://www.macrogen.com). Subsequently, the chromatograms of the sequences were manually edited using BioEdit version 7.2.5 [44] and analysed using the BLAST tool (https://blast.ncbi.nlm.nih.gov/Blast.cgi) to determine sequence identity between the PCR products and the ITS2 sequences of the *An. gambiae* complex.

### Field sample investigations

Morphological identification was conducted on all field samples, which were (mis)identified as belonging to the *An. funestus* group. Species identification was performed on a subset of field samples (n = 28) molecularly identified as *An. leesoni* using the *An. funestus* multiplex PCR [35]. The ITS2 PCR and mDNA cytochrome oxidase I (COI) loci [35, 37] PCR followed by sequencing of the PCR amplicons was used for these species identifications. The resulting sequences were analysed using nBLAST (https://blast.ncbi.nlm.nih.gov/Blast.cgi). In addition, these samples were also amplified using conventional PCR methods for the identification of mosquitoes in the *An. gambiae* complex [34, 45] and *An. moucheti* complex by multiplex PCR assays [46], to rule out the possibility of morphological misidentification at the start.

PCR using the UV and LEES primers of the field samples was performed. *Anopheles gambiae* complex specimens used as controls in the PCR were *An. gambiae* sensu stricto (s.s.) (KISUMU colony)*, An. coluzzii* (AKRON colony)*, An. gambiae/coluzzii* hybrid (ASEMBO colony)*, An. arabiensis* (KGB colony) as well as *An. funestus* (s.s.) (FUMOZ colony). Sequencing analysis was performed on the resultant PCR amplicons of the field samples.

## Results

Intra-laboratory proficiency assessment of the *An. funestus* multiplex PCR assay revealed that *An. arabiensis* DNA amplifies a ~ 150 bp fragment and can therefore be incorrectly identified as *An. leesoni,* which amplifies a fragment of similar size [35]. In silico analyses of the primer sequence similarity revealed a 100% sequence identity of UV to the 3′ region of the 5.8S region flanking the ITS2 region of members of the *An. gambiae* complex (Table 1) as can be expected from this highly conserved region [35]. The species-specific reverse primers shared a variable degree of identity with the *An. gambiae* complex (Table 1). The LEES reverse primer had a 77% sequence identity with the ITS2 region of *An. arabiensis*. It was also the only primer which showed over 50% sequence identity with the ITS2 region of other members of the *An. gambiae* complex in the location 120 to 153 bp downstream of the UV primer binding site, therefore producing an amplicon size diagnostic for *An. leesoni*. Additionally, the LEES primer had the highest number of consecutive bases (7) at the 3′ end that directly bound with the ITS2 region of the *An. gambiae* complex member species (Table 1).

**Table 1:** Sequence analysis between *An. funestus* multiplex PCR primers and the ITS2 region of member species of the *An. gambiae* complex

*An. funestus* multiplex PCR primers	*An. arabiensis* ITS2* sequence		*An. gambiae* ITS2* sequence		*An. merus* ITS2* sequence		An. quadriannulatus* ITS2 sequence	
	% Identity with primer	Number of consecutive bases at 3ʹ end of primer that binds with sequence	% Identity with primer	Number of consecutive bases at 3ʹ end of primer that binds with sequence	% Identity with primer	Number of consecutive bases at 3ʹ end of primer that binds with sequence	% Identity with primer	Number of consecutive bases at 3ʹ end of primer that binds with sequence
UV	100	19	100	19	100	19	100	19
FUN	63	3	63	3	63	3	63	3
VAN	57	3	57	3	57	3	57	3
RIV	34	2	63	1	40	0	40	0
PAR	64	4	64	4	64	4	64	4
RIVLIKE	65	0	65	0	63	0	65	2
LEES	77	7	53	7	53	7	53	7

*The ITS2 sequences of the *An. gambiae* complex species are: *An. arabiensis* ITS2 sequence (KT160245.1, GenBank); *An. gambiae* ITS2 sequence (KT120234.1, GenBank); *An. merus* ITS2 sequence (GQ870313.1, GenBank) and *An. quadriannulatus* ITS2 sequence (JN994146.1, GenBank)

The *An. funestus* multiplex PCR assay was subsequently evaluated on other members of the *An. gambiae* complex, and all species tested produced *An. leesoni* diagnostic PCR product (~ 150 bp, Table 2). The exclusion of LEES primer resulted in no amplification (Table 2) regardless of *An. gambiae* complex species or annealing temperature analysed.

**Table 2:** Summary of the results from the different iterations of the *Anopheles funestus* multiplex PCR used to amplify DNA from members of the *An. gambiae* species complex

Samples used for *An. funestus* multiplex PCR	~ 150 bp amplicon produced via *An. funestus* multiplex PCR	~ 150 bp amplicon produced by *An. funestus* multiplex PCR without LEES primer	~ 150 bp amplicon produced by *An. funestus* multiplex PCR with UV and LEES primers only
*An. funestus*	No—an amplicon of 500 bp was produced, which corresponds to the *An. funestus* amplicon	No—an amplicon of 500 bp was produced, which corresponds to the *An. funestus* amplicon	No
*An. leesoni*	Yes	No	Yes
No template negative control	No	No	No
Extraction kit negative control	No	No	No
*An. arabiensis*	Yes	No	Yes
*An. gambiae*	Yes	No	Yes
*An. merus*	Yes	No	Yes
*An. quadriannulatus*	Yes	No	Yes

**Fig. 1:**
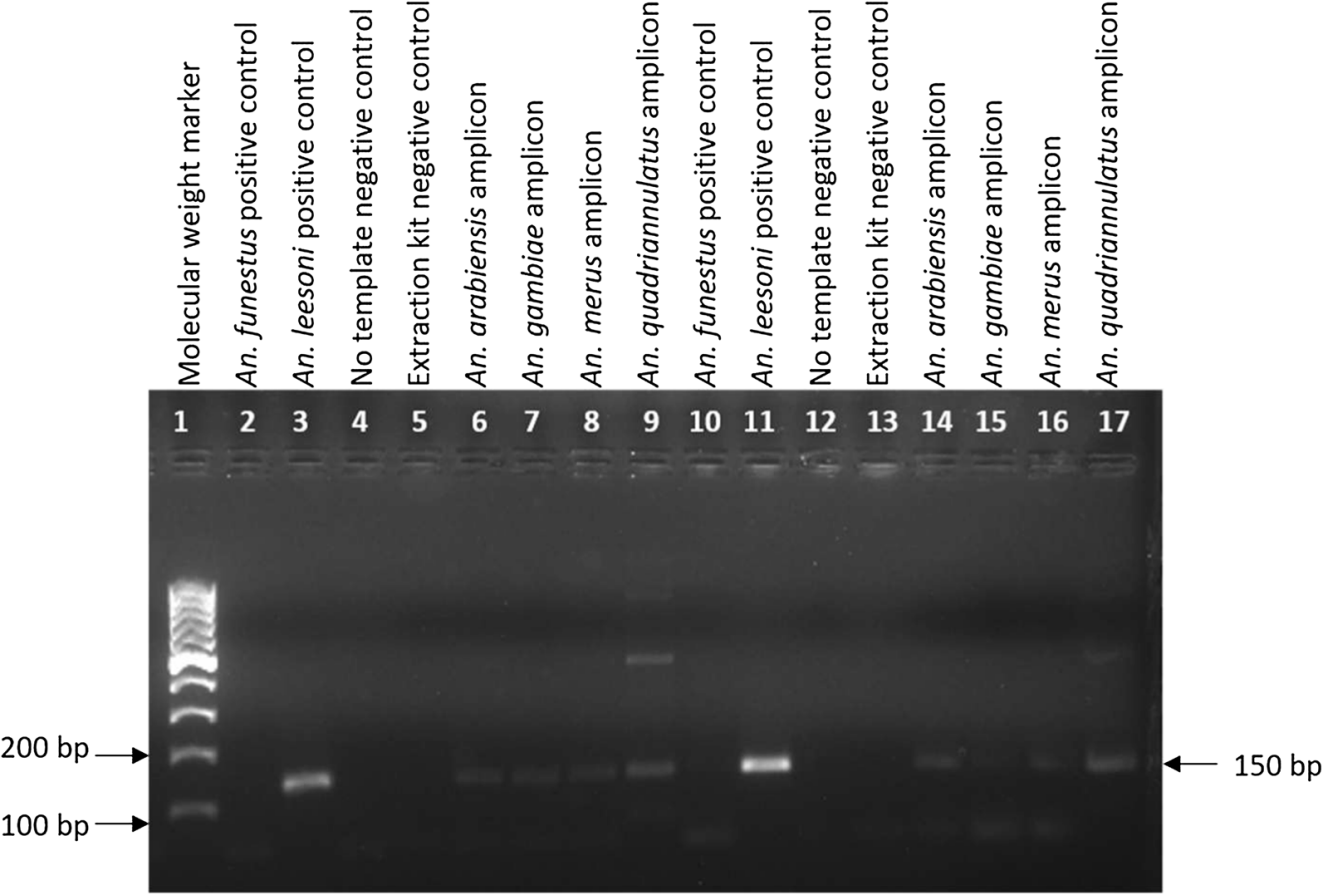
An amplicon of ~ 150 bp (black arrow on the right) was produced when the UV and LEES primers amplified DNA from member species of the *Anopheles gambiae* complex. An additional amplicon of around 500 bp was present in the *An. quadriannulatus* sample. This is most likely due to non-specific binding of the LEES primer to the ITS2 region of *An. quadriannulatus*. The PCR was performed, with annealing temperature set at 45 °C (lanes 2 to 9) or 50 °C (Lanes 10 to 17)

Amplification of DNA from members of the *An. gambiae* complex using only the UV and LEES primers and the *An. funestus* PCR protocol yielded a ~ 150 bp PCR product from all species (Fig. 1; Table 2). Sequence analysis of these PCR amplicons using the UV and LEES primers revealed that there was 99–100% sequence identity between the amplicons and the ITS2 region of *An. gambiae* complex species. Furthermore, sequencing of the PCR amplicons, using UV as the sequencing primer, revealed that the LEES primer sequence was incorporated into the PCR amplicon sequence. This confirms that the LEES and UV primers are responsible for the 150 bp fragment when *An. funestus* PCR is used to amplify the ITS2 of *An. gambiae* complex species, which leads to their misidentification as *An. leesoni*.

### Field sample data

**Fig. 2:**
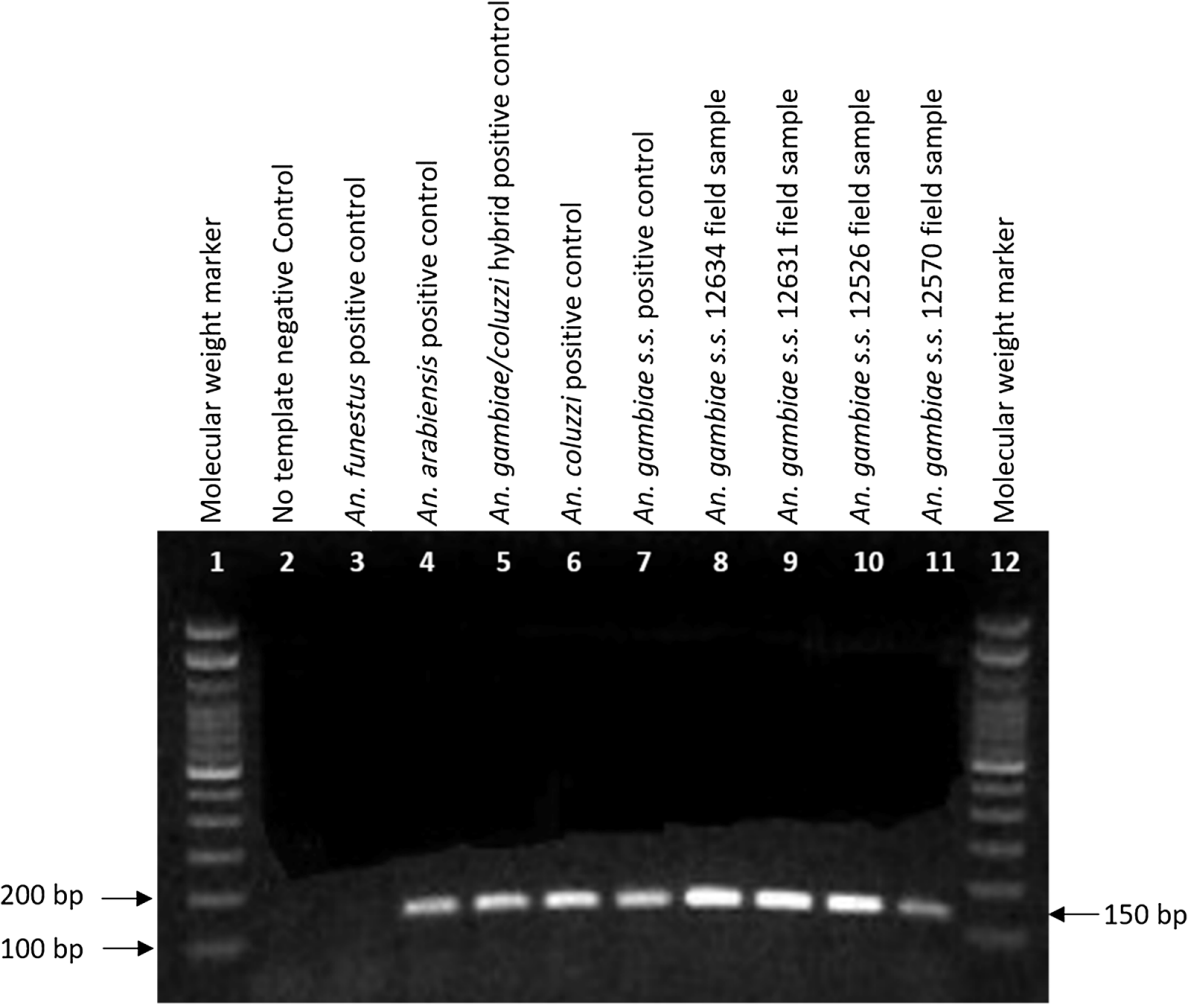
An amplicon of ~ 150 bp (black arrow on the right) was produced when UV and LEES primers amplified DNA from laboratory-reared *An. gambiae* s.s., *An. arabiensis, An. coluzzii, An. gambiae/An coluzzi* hybrid and as well as *An. gambiae* s.s. field samples

A large number of field-collected samples from the Democratic Republic of the Congo were morphologically identified as *An. funestus* group and subsequently molecularly identified as *An. leesoni.* The ITS2 and COI regions were amplified by PCR and sequenced, showing that a subset of these samples were *An. gambiae* s.s. (n = 13) and *An. moucheti* (n = 12). Those identified as *An. gambiae* s.s. through sequencing were further confirmed by *An. gambiae* complex PCR [34, 45]. The samples identified as *An. moucheti* through sequencing were further confirmed by *An. moucheti* multiplex PCR assay [46]. PCR amplification of these samples using the UV and LEES primers produced an *An. leesoni* sized amplicon between 100 and 200 bp. Additionally*, An. gambiae* complex specimens that were used as controls in the PCR—*An. gambiae* s.s., *An. coluzzii, An. gambiae/coluzzii* hybrid and *An. arabiensis*—also produced similar-sized fragments (Fig. 2). Sequencing of the field samples using the UV and LEES primers in the PCR confirmed that the LEES primer fragment was incorporated in the sequences of the PCR amplicons.

### *Anopheles gambiae* multiplex PCR does not amplify DNA from *Anopheles leesoni*

It has been demonstrated that *An. gambiae* complex member species can be misidentified as *An. leesoni* by PCR. In contrast, the *An. gambiae* multiplex PCR does not amplify DNA from *An. leesoni* and cannot therefore misidentify this species as a member of the *An. gambiae* complex.

## Discussion

The importance of correct identification of *Anopheles* species in malaria vector control programmes is critical in terms of choice of control intervention and insecticide product. Accurate species identification enables assessments of vector competence, insecticide susceptibilities and important behavioural characteristics (such as feeding and resting behaviours) by species, leading to the design of coherent insecticide-based control strategies that can be enhanced by additional methodologies for malaria elimination. These data indicate that if members of the *An. gambiae* complex (*An. arabiensis*, *An. gambiae*, *An. coluzzii, An. merus* and *An. quadriannulatus*) as well as *An. moucheti* are morphologically incorrectly identified as *An. funestus* group, they can be falsely identified as *An. leesoni* when using an *An. funestus* multiplex PCR.

This is due to high primer (specifically UV and LEES) sequence identity between the two species groups. The UV primer showed a 100% sequence identity to the ITS2 region of the *An. gambiae* complex. This is not surprising, since the UV primer is in the conserved region of the 5.8S ribosomal RNA gene [35]. The LEES primer sequence identity with the *An. gambiae* complex ITS2 region ranged between 53% and 77%. The likely reason for the amplification of *An. gambiae* complex DNA using the LEES primer is due to the seven consecutive bases at its 3′end. These bases specifically bind to the ITS2 region of the *An. gambiae* complex. In a PCR reaction, this leads to the incorporation of the LEES primer 120 bp downstream of the UV primer binding region to produce an amplicon of the *An. gambiae* complex species ITS2, which had the LEES primer binding region in its sequence as was evidenced by the sequencing data. This is also true for *An. moucheti*. This scenario is expected to be the case in other species of the *An. gambiae* complex, such as *An. coluzzii, Anopheles bwambae* and *Anopheles amharicus*, because the same 7 bases of the LEES primer bind to the ITS2 regions of these species (GenBank Accession numbers: KT160244.1; GQ870320.1 and GQ870316.1). Indeed, an *An. coluzzii* sample that was used as a control in the PCR (using the UV and LEES primers), for the field samples analysis, produced a positive *An. leesoni* sized amplicon band. The fact that *An. gambiae* complex species can be misidentified as *An. leesoni* supports a recent publication by Erlank et al. [33], which demonstrated that *Anopheles rufipes* and *Anopheles rhodesiensis* can misleadingly be identified as *An. leesoni* with the use of *An. funestus* multiplex PCR.

Different *Anopheles* species vary in their malaria vectorial capacities as well as in their feeding and resting habits [11, 47]. They may also have different insecticide susceptibility profiles and, therefore, their correct identification to species is vital for the implementation of an efficient vector control strategy based on accurate vector incrimination and appropriate use of insecticides. Members of the *An. gambiae* complex and *An. funestus* group are often found in sympatry [11, 47, 48]. It is, therefore, likely that the collection of field samples could contain a mix of species, making accurate identification to species essential.

These data also raise concerns over previously published records of vector incrimination of species identified as *An. leesoni* by *An. funestus* multiplex PCR alone, which was common practice at the time [14]. This stresses the importance to confirm species identity through ITS2 and/or COI sequencing to prevent mis-interpretation of data.

There are several steps necessary to minimize the misidentification of species from the *An. gambiae* complex as *An. leesoni*. The first step, which is also highlighted by Erlank et al. [33], is to accurately identify the samples morphologically. However, morphological species identification is largely dependent on the condition of the sample—field-collected samples may be damaged—as well as the skill of personnel involved, the equipment they have and their workload. In the event that a field sample is suspected to be *An. leesoni* via the *An. funestus* multiplex PCR, but the morphological identification is not certain, it is advisable to use *An. gambiae* multiplex PCR on the DNA of the sample. The results from this study indicate that DNA from a true *An. leesoni* sample does not amplify using the *An. gambiae* multiplex PCR, eliminating any uncertainty regarding the identity of the field sample. Additionally, should a suspected *An. leesoni* female test positive for *P. falciparum* sporozoites by ELISA [49] and/or PCR [50, 51], it is necessary to perform an ITS2 and/or COI sequence confirmation of the mosquito sample so as to eliminate any ambiguity regarding vector status [21, 35, 37].

## Conclusions

Member species of the *An. gambiae* complex can be misidentified as *An. leesoni* when analysed using the *An. funestus* group multiplex PCR. This is best avoided by accurate morphological identification prior to PCR assessments and can also be resolved by further analysing samples using the *An. gambiae* multiplex PCR where sequencing technology is not available. Lastly, it is important for the reference laboratory performing species identifications to periodically conduct quality control assessments and proficiency testing of laboratory personnel. Sequence analysis should be performed to confirm the species identity in cases of conflicting results. This ensures that the correct species identifications are reported to malaria vector control programmes.

## Data Availability

The datasets used and/or analysed during the current study are available from the corresponding author on reasonable request.

## Abbreviations

ATSB: Attractive toxic sugar baits
BLAST: Basic Local Alignment Search Tool
COI: Cytochrome oxidase I
ELISA: Enzyme-linked immunosorbent assay
FUN: *An. funestus* species-specific reverse primer
IRS: Indoor residual spraying
ITN: Insecticide treated nets
ITS2: Internal transcribed spacer 2
LEES: *An. leesoni* species-specific reverse primer
LSM: Larval source management
NICD: National Institute for Communicable Diseases
PAR: *An. parensis* species-specific reverse primer
PCR: Polymerase chain reaction
QA: Quality assurance
RIV: *An. rivulorum* species-specific reverse primer
RIVLIKE: *An. rivulorum*-*like* species specific reverse primer
UV: Universal forward primer
VAN: *An. vaneedeni* species-specific reverse primer
WHO: World Health Organization
